# The Impact of Cognitive Load on the Cardiac Orienting Response to Auditory Structural Features during Natural Radio Listening Situations

**DOI:** 10.5334/joc.43

**Published:** 2018-07-31

**Authors:** Robert F. Potter, Joshua Sites, Edgar Jamison-Koenig, Xia Zheng

**Affiliations:** 1 The Media School, Indiana University, Bloomington, US

**Keywords:** Audio, attention, orienting response, habituation, radio

## Abstract

Previous research has shown that structural features such as voice changes, jingle onsets, and production effects in a radio broadcast elicit cardiac orienting responses. In fact, the voice change has been shown to reliably elicit orienting without habituation after several repetitions. However, repeated onsets of two other auditory structural features—jingles and production effects—did result in habituation when the participant was exposed to them embedded in an audio production absent a central cognitive task. This article presents two experiments testing the possibility that adding a central task prevents the development of a robust neural model of the auditory structural features necessary for habituation. In both studies, results show that adding a primary cognitive task eliminated habituation to jingles and production effects. However, varying the cognitive load of the primary task across two levels of difficulty had no significant effect on habituation.

For decades, audio producers have instinctively used certain techniques to increase the likelihood of gaining the listener’s attention. This has resulted in an accepted set of techniques or “tools of the trade” believed to be able to cut through a cluttered auditory landscape resulting in greater listener attention and more effective productions. Sound effects, music, and vocal exclamations are often intuitively employed in such a manner by professional audio producers. In support of this routine industry practice, a substantial literature now exists that uses physiological and behavioral data to demonstrate the ability of auditory structural features to increase automatic attention capture among listeners (Potter, 2000; Potter, Lang & Bolls, 2008). Much of this literature is based on predictions concerning the phenomenon known as the orienting response (OR; Sokolov, 1963; Öhman, 1979). The OR is an automatic allocation of cognitive resources to encoding of information from the environment (Graham, 1979). Sometimes, this allocation is driven by a learned signal—such as the cocktail party effect where one experiences their name ‘pop out’ from an otherwise inaudible conversation going on across a crowded room (Cherry, 1953). More frequently, however, the OR occurs following the onset of novel information in the environment (Graham, 1979). Functionally, the OR is thought to have evolved due to benefits derived from a rapid and automatic evaluation of new information and the resulting activation of a defensive motivational system as protection, or an appetitive system in response to beneficial presentations of food and potential mates.

There are many ways to identify an OR, the most obvious being the turning of the perceptual organs in the direction of the signal or novelty (Pavlov, 1927). This is a possible operational definition when trying to assess orienting in a generally inattentive crowd of people, for example. However, it is less helpful when examining the ORs that occur when a subject is already turned in the direction in which the novelty is expected to occur. This is commonly the case in experiments designed to determine whether certain aspects of media messages result in an OR. In television, for example, the viewer is often looking right at the video monitor, making it nearly impossible to use spatial orientation of the head to determine when the OR has been elicited. Using bodily positioning to answer questions about automatic attention capture by audio is also complicated by the delivery of sound stimuli by headphones and the overall background nature of many environmental sounds. In situations such as these, physiological measures can be enlisted to help identify orienting. These include both temporal based EEG measures such as the ERP (Graham, 1992; Näätänen, 1992) and frequency based brain wave analyses such as alpha blocking (Lynn, 1966; Becker & Shapiro, 1981; Reeves, Thorson, & Schleuder, 1986). Orienting also can be indexed by pupil dilation (Lynn, 1966), increases in skin conductance on the palm (Öhman, 1979; Potter et al., 2008), and specific patterns in the cardiac response (Graham, 1979; Lacey & Lacey, 1974). In heart rate, the OR is identified through a significant deceleration in response to the evoking stimuli, forming either a U-shaped (quadratic) or S-shaped (cubic) pattern in the 6–10s following the onset of the eliciting stimulus (Chase & Graham, 1967).

Media psychology research has used cardiac orienting to identify automatic attention allocation to sources of novelty in the mediated environment such as camera changes (Lang, 1990), informational text onsets (Thorson & Lang, 1992), and web animation (Diao & Sundar, 2004). In audio, Potter (2000) showed that the structural feature of the voice change elicited cubic cardiac deceleration in a 6–10s window following onset. A voice change, conceptualized as the instantaneous replacement of one speaker’s voice by another, regardless of gender, was selected to initially study orienting to auditory structural features because of its analog with the camera change in video messages (Lang, 1990; Lang, Geiger, Strickwerda, & Sumner, 1993). Both voice changes and camera changes are used to move a story along in audio and video, respectively. Potter et al. (2008) later demonstrated that the onset of several auditory structural features result in cardiac deceleration in the quadratic or cubic pattern associated with orienting (Graham, 1979). These include sound effects, production effects (e.g. laser and drone sounds common in modern radio), and jingles (i.e. musical station identifications).

A common phenomenon associated with the OR is habituation, a diminishment and possible eventual extinction of the automatic response after the stimulus occurs repeatedly (Graham, 1979). There are conflicting findings in the literature concerning whether humans habituate to repetitive background sounds; some results suggest they do (Banbury & Berry, 1997; Bell, Röer, Dentale, & Buchner, 2012; Morris & Jones, 1990) and others do not (Röer, Bell, Dentale, & Buchner, 2011). Across most of these studies, however, is a common conceptualization of the OR resulting from comparisons made between neural models of the environment over time (Öhman, 1979; Cowan, 1995). Orienting occurs when the model of previously encountered stimuli does not match that created via perception of the current experience. However, after multiple stimuli onsets, the compared neural models begin to overlap and the OR begins to diminish.

Using the conceptualization of a neural model as a way to think about the processing of structural features in media messages, it is logical to predict that following exposure to multiple camera changes in a 30-second television advertisement, for example, the neural model of the perceptual phenomenon might contain the occurrence of a camera change and habituation of the cardiac OR would result. Interestingly, this does not seem to be the case in video camera changes, which continue to elicit orienting after many repetitions (Lang, 2006; Lee & Lang, 2015). Similarly, Potter (2000) demonstrated that orienting to voice changes in audio messages failed to habituate after six occurrences in 2-minute radio dialogs. Both Potter (2000) and Lang (2000; Lee & Lang, 2015) instructed participants to pay attention to the media message for subsequent memory tests. Each have suggested that the lack of habituation to these media structural feature onsets may be due to the novelty and importance of the information being introduced by the feature, even though the structural feature itself is repetitive. Such an explanation is in line with Bell et al. (2012), who describe habituation of the OR as being a process that “serves to ensure that stimuli that have already been identified as irrelevant for the individual’s goals do not consume limited processing resources” (pg. 1542). Because voice changes and camera changes predictably introduce new information to the media audience—information necessary to the development of a narrative or a conversation—they never become “identified as irrelevant” within the context of the message being communicated.

Other recent research, however, suggests that orienting to other auditory structural features may be different than to the voice change. Potter, Lynch, and Krause (2015) investigated the impact of repetition of station jingles and station identifications produced with laser or drone effects on habituation of the cardiac OR. In two experiments they interspersed 6s repetitions of station jingles and production effects between songs in simulated “Top 40”-style radio broadcasts and measured participants’ cardiac activity following onsets. Participants were told to listen to the broadcast because they would be asked questions about the music afterwards. Statistically significant interactions in the cardiac response curves showed that—unlike voice changes—listeners habituated to onsets of jingles and production effects. In response to the first instances, heart rate decelerated in the cubic shape of an OR, suggesting listener reactions were primarily driven by the parasympathetic nervous system (PNS) and its role of facilitating information intake. This response habituated by the third onset of the production effect stimulus, however. Furthermore, in response to the third occurrence of a jingle stimulus, heart rate accelerated in a manner associated with the fight-or-flight function of the sympathetic nervous system (SNS). Potter et al. (2015) interpreted this as an aversive response to the repeated jingles, although they did not present physiological or self-report data to support such an interpretation.

Given the lack of habituation found in the media psychology literature to camera changes (Lang, 2000; Lang & Lee, 2015) and voice changes (Potter, 2000), the results of Potter et al. (2015) were unexpected. One possibility for the findings, however, is that listeners to the music radio presentation in Potter et al. (2015) were not provided with another task on which to focus central processing resources and therefore had ample working memory to devote to the creation of neural models of the broadcast. The experimental protocol called for participants to sit in a comfortable chair, alone in a room, while listening to the simulated radio broadcast through headphones. Unlike the common procedures of many basic psychology experiments on the irrelevant sound effect—whereby participant’s serial recall of presented information (typically of visual origin) is invariably impaired by the presentation of task-irrelevant background sound (Salamé & Baddeley, 1982; Hughes, Hurlstone, Marsh, Vachon, & Jones, 2013)—participants were not told to ignore the audio presented. However, they were also not instructed to pay particularly close attention to it; participants were just directed to “sit and listen to the radio” for the 45-minute session, and that afterwards they would be asked their attitudes toward the music played. Previous research has shown that active listening to auditory stimuli prior to ignoring it, yields greater habituation effects (Bell, Dentale, Buchner, & Mayr, 2010; Bell et al., 2012). Furthermore, Sörqvist, Nöstl, & Hailin (2012) used response latency to visual targets preceded by either standard or deviant tones to demonstrate that individual differences in working memory were positively correlated with habituation to oddball audio probes. This adds support for the possibility that with available working memory, participants in Potter et al. (2015) developed neural models representing repeated onsets of station jingles and station identifications as providing irrelevant information—with habituation being the result. This would be unlike the neural models generated by camera changes (Lang, 2000, Lee & Lang, 2006) and voice changes (Potter, 2000), where the structural features introduced information advancing the storyline and dialog, and habituation did not occur.

To test this possible interpretation, the principal goal of the current pair of studies is to empirically investigate whether providing participants with a primary cognitive task impacts the habituation of cardiac orienting responses to the onset of station jingles and station identifications created with production effects over the course of a radio broadcast. Such investigation not only adds to the applied cognitive psychology literature but also to that of media psychology. Both studies reported here were designed to increase the ecological validity of the findings in comparison to Potter et al. (2015), since people do not regularly sit for long periods of time listening to “Top 40”-style radio without doing some other activity or task (McDonald & Meng, 2009; McClung, Pompper, & Kinnally, 2007).

## Study 1: Orienting to structural features occurring in natural listening conditions

While recent research suggests that auditory structural features such as station identification jingles and production effects may habituate during listening situations without a central task (Potter et al., 2015), it is possible that this experimental situation allowed participants to actively listen to the stimulus radio broadcast, and particularly the structural features of interest, resulting in greater habituation effects (Bell et al., 2010; Bell et al., 2012). Furthermore, cognitive science literature indicates that a central activity such as a serial recall task may impact how listeners are distracted by background audio information (Hughes et al., 2013; also see Sörqvist & Marsh, 2015). Study 1 explored whether providing a primary task may prevent habituation from occurring by occupying working memory to such an extent that less complete neural models of the background audio could be formed. The specific primary task in this experiment was left up to the participant in order to best replicate a natural listening situation. However, it was not expected that participants would engage in any tasks too cognitively difficult during their experimental session. Since performance in a primary task has shown to be diminished in response to the background audio information under low task difficulty (Hughes et al., 2013), we expected that when participants focus their central processing on a self-selected task they would be less likely to form complete neural models of the background audio. Therefore, the onset of the jingles and production effects would be more likely to result in orienting. In other words, if listeners are involved in a self-directed primary task, orienting responses to auditory structural features in background radio messages will not habituate.

### Method

**Participants.** Students (n = 27) at a large Midwestern university in the United States participated for course credit. Each was told that they would spend about an hour in a research lab and could do anything they wanted during that time except listen to something other than the simulated radio broadcast played through circumaural headphones. Participants were given examples during recruitment of the types of likely activities: surfing the web, texting or using their smart phone, reading, or studying. Upon arrival at the lab each provided informed consent, was randomly assigned to an order of presentation and again given instructions about possible primary tasks. No participants merely sat and listened to the music in a manner similar to Potter et al. (2015).

**Materials/apparatus.** To create the stimuli, popular songs were selected and arranged in four systematic orders to resemble typical commercial radio in the United States. Interspersed between each song (approximately every 4 minutes) were alternating instances of the auditory structural features. The order of structural features was counterbalanced across the four stimuli orders. The structural features were actual radio station identification messages (e.g. “Today’s hits and yesterday’s favorites on Power 95”) in the form of either jingles or production effects. Five different examples of each structural feature were used, each about 6s in duration. Total running time of the completed stimuli was 45:30.

Physiological data were collected at a sampling rate of 1000 Hz by a Biopac MP150 system. Acqknowledge software calculated heart rate data in bpm/sec based on the interbeat intervals of the QRS-complex in the electrocardiogram. The experiment was controlled by MediaLab software (Jarvis, 2016) and time locked to the physiological data via TTL signal transmitted via parallel port.

**Design.** A 2 (Structural Feature Type) × 5 (Repetition) within-subjects factorial design was employed. Structural Feature Type had two levels – Jingles and Production Effects – with five Repetitions of different examples for each.

**Procedures.** Five electrodes were attached to each participant’s skin. Two AG/AGCL electrodes filled with non-conductive gel were attached to the palm of the participant’s non-dominant hand to avoid interference with the regular use of a computer mouse. These electrodes were used to measure electrodermal activity (EDA), data which are not reported here.

Three floating AG/AGCL electrodes were attached to the inside of the participant’s forearms to collect heart rate (HR) data. Prior to placing these electrodes, the surface of the skin was wiped clean with a mildly abrasive alcohol prep pad.

Participants were instructed to do whatever they wanted during the lab session while listening to the radio stimulus through headphones. Suggestions provided during recruitment were repeated and included reading, surfing the web, studying, or using their mobile phones to access apps or text. The only requirements were that they could not take off the headphones and they must remain seated to ensure that the physiological sensors remained in place.

Following the presentation of the stimuli, the physiological electrodes were removed and participants were played short segments of each of the songs heard during the radio stimuli. Self-report data were collected about their familiarity with, and attitudes toward the songs. Those data are not reported here.

**Data cleaning, preparation, and analysis.** The onset of each jingle and production effect was located in the physiological data and visual inspection of the peaks of the QRS-complex identified by the Acqknowledge software were conducted for the 5-seconds prior and 10-seconds after each onset. This ensured clean cardiac data within a sufficient baseline period and the window following feature onset in which ORs were expected. The milliseconds between each peak were then used by the Acqknowledge software to calculate a weighted average for each 1-second time increment following onset. Change scores were calculated in Excel from the feature onset for each of the subsequent 5-seconds (Potter & Bolls, 2012).

The hypothesis was tested by submitting the cardiac change score data to a 2 (Feature Type) × 5 (Repetition) × 6 (Time) repeated measures ANOVA. Significant quadratic or cubic trends in the shape of the overall cardiac response curve (CRC) would demonstrate the occurrence of an OR (Graham, 1979). All *p* values for psychophysiological analyses are presented after Huynh-Feldt adjustments to the degrees of freedom due to the violation of sphericity common in psychophysiological data.

### Results

**Figure 1:**
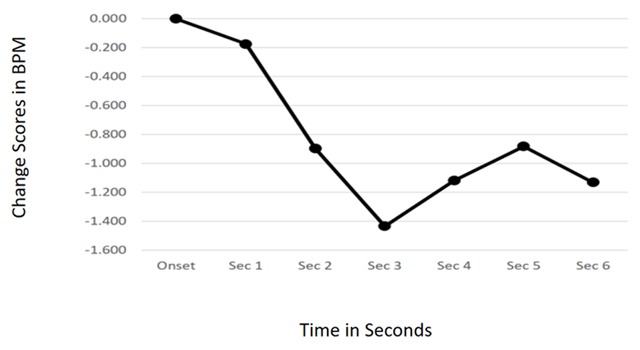
Cardiac Response Curve showing orienting to auditory structural features in Study 1, collapsed across Feature Type and Repetition.

Anecdotal observations by those collecting the data suggest that most participants listened to the audio stimulus while texting people and surfing the internet on their mobile phone. The prediction was that if listeners were involved in a self-directed primary task, orienting responses to the onset of station jingles and production effects would occur and not habituate following repetition of the feature type. When collapsing across all Repetitions and Structural Feature Types, there was a main effect of Time, *F*(6, 144) = 6.174, *p* = 0.001. The quadratic trend was also significant, *F*(1, 24) = 7.486, *p* = 0.012, indicating orienting to the structural features. The CRC can be seen in Figure 1.

**Figure 2:**
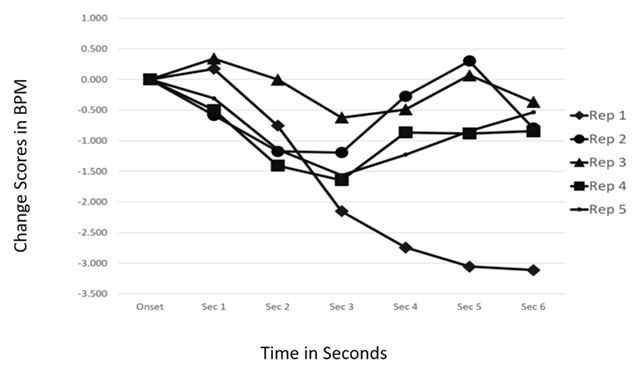
Time × Repetition interaction, n.s. Orienting occurred in response to each repetition of structural feature in Study 1.

The F-value for the Structural Feature Type × Repetition × Time interaction was < 1. Additionally, the Repetition × Time interaction was not statistically significant, *F*(24, 576) = 1.485, *p* = 0.17. This can be seen in the multiple CRCs shown in Figure 2. Although the first repetition is qualitatively different than the others, inspection of the CRC beyond the typical 6-second window used to identify ORs showed that the orienting to the first onset took longer to return to baseline, likely due to the heightened novelty of a first occurrence. However, all five CRCs show cardiac decelerations and not eventual non-response or acceleration as demonstrated in Potter et al. (2015). This leads to the conclusion that ORs were elicited by all five repetitions of the auditory structural features when the participants were focused on a simple primary task of their own choosing. The hypothesis is supported.

## Study 2: The impact of cognitive load on orienting to repeating audio structural features

Study 1 showed that a simple self-selected primary task eliminates habituation to auditory structural features found during a passive listening protocol (Potter et al., 2015). However, Study 1 provided little control over the cognitive load incurred by the participant’s primary task. The goal of Study 2, therefore, was to systematically vary the working memory load of the task while still providing a natural radio listening environment for participants. This was done by tasking them with playing a computer word game set at either the easy or hard level of difficulty.

Much of the work investigating the impact of working memory load on the magnitude of the irrelevant sound effect used ERP measures as the physiological dependent variable (SanMiguel, Corral, & Escera, 2008; Berti & Schröger, 2003). There are three discernable ERP components associated with the OR (Berti & Schröger, 2003). The first is the Mismatch Negativity (MMN) which is a pre-attentive response to discrepancies between the neural model of the environment stored in working memory and the one created through perceptual intake (Näätänen, 1992). The second ERP component, the Novelty P3, is associated with triggering controlled resource allocation to processing the source of novelty (Graham, 1992). The Reorienting Negativity (RON) occurs approximately 500 ms from the stimulus onset and is thought to correspond to attention shifts back to the primary task (Sussman, Winkler, & Schröger, 2003). It is a robust finding that differences in working memory load do not impact the MMN, but instead attenuate the strength of the late stages of the P3 in situations of higher resource load (San Miguel et al., 2008; Berti & Schröger, 2003). Attempts to synthesize theories of attention allocation across researchers who use ERP and those who use peripheral nervous system measures such as heart rate have been difficult due to differences in temporal units of analysis (Donchin, et al., 1984). Perhaps for this reason, it is rare for studies to use both EEG and HR as indications of ORs. One study (Simons & Perlstein, 1997) reports a correlation between P3 amplitude and cardiac change scores associated with orienting to auditory probes. This suggests that, given decreased P3 in conditions of greater working memory load, a smaller impact on cardiac orienting would be expected as well. This corresponds with results from Thorson and Lang (1992) who measured cardiac ORs in response to the onset of text information in educational videos and found diminished responses when the instructional content was difficult than when it was familiar. Given this, the following is hypothesized: There will be a Task Difficulty × Time interaction on the cardiac change score data such that during the High Task Difficulty trial participants will have less pronounced ORs than during the Low Task Difficulty trial.

With the expectation that less resources will be available to process the audio stimulus during the difficult version of the word game than the easy, it is also predicted that a less complete neural model of the structural features will be created. This will result in a decreased likelihood that habituation to the feature onsets will occur during high load than during low load: There will be a Task Difficulty × Repetition × Time interaction on the cardiac change score data such that during the Low Task Difficulty trial the OR will habituate but during the High Task Difficulty trial it will not.

### Method

**Participants.** Fifty-nine participants (Males = 23) completed Study 2 after being recruited from the same large Midwestern university. All participated for course credit and provided informed consent.

**Materials/apparatus.** Although the general construction of the stimuli matched Study 1, having the task-difficulty remain a within-subjects variable required two versions of simulated radio to be created for each participant. One version had production effects interspersed between the songs, and the other version had jingles interspersed. To prevent the data collection session from being too long given the within-subjects nature of the Task Difficulty factor, each structural feature repeated three times rather than the five repetitions used in Study 1. No song was repeated across the two versions. The order of music and structural features in both versions remained constant across participants, but the pairing of the two types of structural features was counterbalanced across both levels of Task Difficulty. Physiological data were collected and time locked to the stimulus in the same manner as in Study 1.

**Figure 3:**
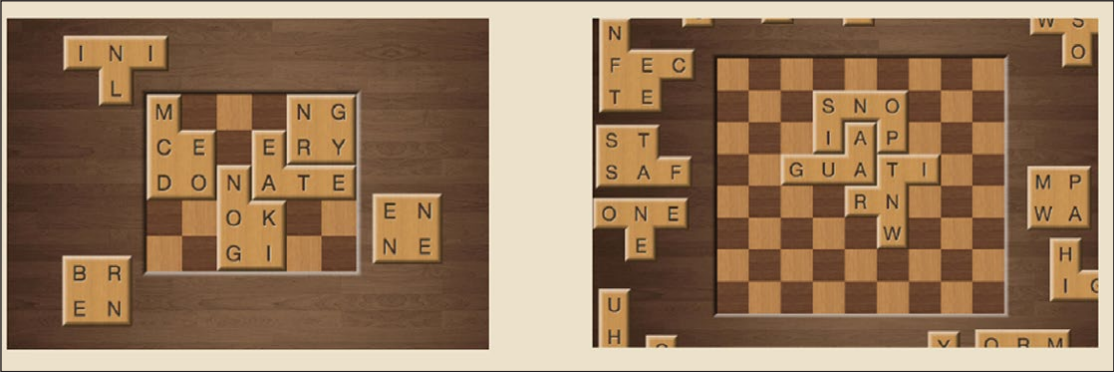
Examples of the Easy (left) and Hard (right) levels of the Task Difficulty factor. From http://www.wordjigsaw.com/.

**Design.** The study used a 2 (Task Difficulty) × 2 (Structural Feature Type) × 3 (Repetition) within-subjects factorial design. While listening to the radio stimulus, participants played the Word Jigsaw game (Pierce, 2016) on a computer for fifteen minutes. The game presented a checkers-like board to the players of either 6 × 5 squares (Low Task Difficulty) or 9 × 8 squares (High Task Difficulty). Along the outside of the board were preset tiles containing two and/or three-letter sequences. The tiles fit together only one way on the board such that all the letter sequences formed English words (See Figure 3).

**Procedures.** The same electrode application procedures used in Study 1 were followed. Then the instructions for the word puzzle game were explained, along with a description of a monetary incentive. Participants were motivated to work dutifully at the word game by earning entries into a random drawing for a $50 USD gift card with each successful completion of the game. Each word game completed on the Low Difficulty earned participants one entry and each game completed on the High Difficulty earned five entries. This was done to encourage continued engagement in the High Difficulty trial.

Following the two sections of gameplay, the physiological electrodes were removed from the participants and they completed a forced-choice recognition task, data from which is not reported here. The duration of the experiment was approximately 45 minutes per participant.

### Results

**Figure 4:**
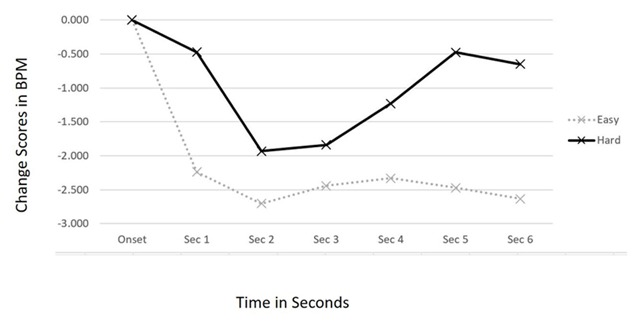
Cardiac Response Curves showing orienting to auditory structural features across levels of Task Difficulty.

We predicted that placing a greater cognitive load on participants through a harder primary task would diminish the orienting response to the onset of jingles and production effects in the background radio stimulus. As a manipulation check on this cognitive load assumption, we noted the number of word puzzles that participants were able to complete during each trial. Significantly fewer games were finished in the Hard Task Difficulty condition (*M* = 0.12) than in the Simple Task Difficulty condition (*M* = 3.78), *t*(59) = 7.07, *p* < 0.001. Given this, we could move to test the Task Difficulty × Time interaction on the cardiac change scores. Results show this interaction was not statistically significant, *F*(6, 342) = 1.680, *p* = 0.161. However, the main effect of Task Difficulty was marginally significant, *F*(1, 57) = 2.718, *p* = 0.105. As seen in Figure 4, the dynamic patterns are similar but as predicted the cardiac deceleration was attenuated during the higher load trials.

**Figure 5:**
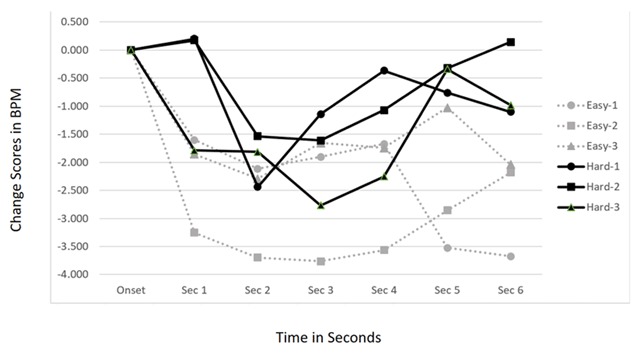
Cardiac Response Curves showing repeated orienting to auditory structural features across levels of Task Difficulty.

We also predicted that habituation to the structural features would be less likely during times of high cognitive load given that the primary task would prevent a robust neural model of the background audio from being formed. The 2 (Task Difficulty) × 3 (Repetition) × 6 (Time) interaction was not significant, *F*(12, 684) < 1, *p* = 0.677. As can be seen in Figure 5, there was robust orienting to all three repetitions of the structural features in both the Hard and the Easy trials.

## Discussion

Potter et al. (2015) demonstrated that station identifications in the form of jingles and production effects cause automatic attention capture, but that the OR to them eventually habituates. It is possible that the experimental design of Potter et al. (2015), which did not give participants a central cognitive task, resulted in ample available resources for participants to develop a robust neural model of the audio environment. Such a model likely categorized the onset of jingles and production effects as irrelevant to current goals, which precipitated the eventual habituation to their onsets. This article presented two studies designed to test this possibility.

In Study 1, participants were allowed to select their own primary task, provided they could do it while seated and wearing headphones in order to hear the simulated radio broadcast. Results support the idea that when primary resources were even casually devoted to something other than the audio stimulus, habituation of the orienting responses to structural feature onset was eliminated. Cardiac response curves following the onset of jingles and production effects were nearly iconic representations of ORs in all cases but one (Rep 1, Figure 2). Even in that case, the response is one of sustained cardiac deceleration suggesting substantial parasympathetic activation—and the allocation of cognitive resources to new information in the environment. So, the results of the first study add support to the idea that attention capture by irrelevant sounds is at least somewhat influenced by the levels of controlled processing that the individual is devoting to a central task.

In Study 2 we systematically varied the difficulty of the task by having participants play both a simple version and a difficult version of a computer word puzzle game. It was predicted that the comparative increase in the resource allocation to the difficult version of the game would result in less pronounced cardiac orienting to the auditory structural features compared to the ORs occurring during the simple version of the game. Although the general pattern supported this prediction (Figure 4), the data failed to reach statistical significance. We also predicted that, given that participants would have more working memory available to form accurate neural models of the audio features during the easy word puzzles compared to the hard ones, more robust neural models of the background audio would be formed and habituation would be more likely to result. This was also not supported by the data to a level of statistical significance.

There are several reasons why the predictions in Study 2 may not have been confirmed. One is that most studies used to support the first hypothesis utilized ERP results to identify the impact of working memory on the physiological aspect of orienting responses (SanMiguel, et al., 2008; Berti & Schröger, 2003). There is only limited evidence linking the rapid response of the P300 element of orienting to changes in background sound and the cardiac response (Graham, 1992; Simons & Perlstein, 1997). It is quite possible that the P300 differences found in response to irrelevant sounds happen so quickly after onset and continues for such a brief time that the cardiac deceleration initiated by the MMN—which remains unaltered by differences in working memory (Graham, 1992)—is too large and therefore the heart is an insensitive organ with which to identify these differences.

Other studies have used memory performance to suggest that increasing top-down memory load attenuates the distractibility of background deviant sounds (Hughes, et al., 2013). For example, in their study, Hughes et al. (2013) demonstrated that the disruption that a voice deviant produced to serial recall was eliminated when participants were required to recall stimuli that were masked with visual noise (masked digit-sequence memory task). Future research may wish to consider using ERP and/or memory performance as dependent variables to investigate the question of habituation to structural features in radio under different cognitive loads. The benefit of the current set of studies—namely the high external validity in the primary task—may need to be sacrificed in order to do so. The task we adopted for participants in Study 2 was playing a video game, something that conceivably many people do while listening to background radio. This is not the case for the digit and masked-digit sequence memory task of Hughes et al. (2013). Similarly, on the stimulus side, our radio broadcast was designed to simulate what listeners would find in real life: songs interspersed with station identification messages that served as the structural features of interest. Having such a stimulus delivery format only allowed the structural features in Study 2 to be repeated three times. The noise associated with the ERP signal would require many more repetitions of the structural feature in order to find an effect; San Miguel et al. (2008) used 1000 trials divided into four blocks, for example. These experimental design challenges will need to be overcome in future studies in order to continue testing whether the level of cognitive load impacts habituation to the radio structural features jingles and production effects.

Another possible impact on our findings in Study 2 was the nature of our task. By choosing a word puzzle game, we presented a very specific kind of cognitive load, one that required solving semantic and spatial problems which is quite different than trying to remember numeric sequences as is the case with serial recall. Furthermore, the introduction of difficulty seems to be qualitatively different in our study compared to Hughes et al. (2013). Whereas Hughes et al. (2013) introduced a perceptual difficulty and sensory load, the difficulty manipulated within our study mapped onto cognitive difficulty and therefore cognitive load. Increases in sensory load and cognitive load are generally thought to increase susceptibility to distraction (Lavie & de Fockert, 2003, 2005; but see Halin, Marsh, & Sörqvist, 2015) which is generally consistent with the current findings that engaging with a task relative to active listening diminished habituation to the onset of jingles and production effects. We also introduced a different motivation across our high and low difficulty levels of the Task Difficulty factor by providing more entries into the random prize drawing for each completed puzzle in those trials. Although our reasoning was to keep participants engaged in this difficult task, the added motivation may have impacted our results. For example, Ball, Threadgold, Solowiej, and Marsh (2018) demonstrated that motivation can impact the degree to which background speech impairs verbally-based problem solving and it is possible, therefore, that inducing motivation may have diminished the effectiveness of the task-difficulty manipulation in modulating distraction through top-down control (cf. Ball et al., 2018; Hughes et al., 2013).

Another possible impact is the possible variance in how much attention participants devoted to the central tasks as opposed to the stimulus messages—variance which we did not measure. True, the manipulation check indicates that the levels of difficulty effectively lowered the participants’ ability to complete the task—with no participant completing more than one round of the game when it was set to the hard level of task difficulty. We are left to assume the willingness of the participant to remain engaged in the primary task through the end of the high load trial. Although we introduced a monetary incentive to optimize the likelihood, absent a memory test for the content included in the central task to ensure they were remaining engaged, we are left uncertain that all did. Future research should try to establish a way to determine the extent to which engagement continued throughout the high levels of task difficulty. Doing so will not only help to replicate the findings here (which are based on an assumption of engagement), but will also allow to test the hypotheses of repetition on any participants who do *not* remain fully engaged. Those who begin a difficult primary task focused but then disengage would have more working memory available to form neural models of the background audio as the experiment went on, compared to those who continue working hard on the primary task. Therefore, the former group would be more likely to habituate to the onset of jingles and production effects than those whose working memory remain allocated to the central task.

Even given these design-based difficulties in interpreting the data from Study 2, we believe this article presents a step forward in the understanding of the processing of background sounds and how to study it moving forward. Firstly, by combining the results of these two experiments with those reported in Potter et al. (2015) we have a better understanding of the interplay between controlled attention allocation to a central task, the automatic response to changes in the background auditory environment, and the habituation of that response. Secondly, results from Study 2 suggest that while cardiac orienting may be sufficient to identify the occurrence of pre-attentive assessments of auditory differences in the environment—similar to the MMN, the use of heart rate to test hypotheses about the impact of differences in central cognitive tasks on auditory automatic attention capture may not be adequately sensitive. Finally, this article as a whole provides a bridge between literatures in the areas of mainstream cognitive psychology, media psychology and psychophysiology as they apply to the impact of background sound on attention, cognition, and physiological process.

## Data Availability

Data and analysis files may be downloaded here: https://doi.org/10.17605/OSF.IO/5FDPK.
